# Playing With Fire: Proinflammatory Virulence Mechanisms of Group A *Streptococcus*

**DOI:** 10.3389/fcimb.2021.704099

**Published:** 2021-07-06

**Authors:** Shyra Wilde, Anders F. Johnson, Christopher N. LaRock

**Affiliations:** ^1^ Microbiology and Molecular Genetics Program, Graduate Division of Biological and Biomedical Sciences, Laney Graduate School, Emory University, Atlanta, GA, United States; ^2^ Department of Microbiology and Immunology, Division of Infectious Diseases, Department of Medicine, and Antibiotic Resistance Center, Emory University School of Medicine, Atlanta, GA, United States

**Keywords:** Group A *Streptococcus*, *Streptococcus pyogenes*, inflammation, virulence factors, pathogenesis, toxins

## Abstract

Group A *Streptococcus* is an obligate human pathogen that is a major cause of infectious morbidity and mortality. It has a natural tropism for the oropharynx and skin, where it causes infections with excessive inflammation due to its expression of proinflammatory toxins and other virulence factors. Inflammation directly contributes to the severity of invasive infections, toxic shock syndrome, and the induction of severe post-infection autoimmune disease caused by autoreactive antibodies. This review discusses what is known about how the virulence factors of Group A *Streptococcus* induce inflammation and how this inflammation can promote disease. Understanding of streptococcal pathogenesis and the role of hyper-immune activation during infection may provide new therapeutic targets to treat the often-fatal outcome of severe disease.

## Introduction

*Streptococcus pyogenes* (group A *Streptococcus*; GAS) is an exclusively human pathogen (Walker et al., 2014). GAS specifically colonizes the upper respiratory mucosa, primarily the oropharynx, and tonsils and other associated lymphoid tissues as their primary site for carriage, dissemination to other body sites, and transmission between individuals. Humans, particularly children, are commonly transiently colonized without overt symptoms. Infection can be relatively mild (e.g., ‘strep throat’ pharyngitis, impetigo) or serious (including scarlet fever, puerperal sepsis, bacteremia, streptococcal toxic shock syndrome (STSS), necrotizing fasciitis, endocarditis and pneumonia) and result in post-infection immune sequelae (including acute poststreptococcal glomerulonephritis, rheumatic fever, rheumatic heart disease, and Sydenham chorea). GAS is a major health burden and the cause of an estimated 600,000 annual deaths due to these diseases (Ralph and Carapetis, 2012).

The inflammatory symptoms of infection (fever, redness, and swelling) ultimately coordinate antimicrobial processes to act against the pathogen and resolve infection. Successful bacterial pathogens commonly work to avoid immune recognition and delay or prevent their clearance by antimicrobial immune effectors. Strategies include modifying pathogen-associated molecular pattern (PAMP) agonists of Nod-like receptor (NLR) or Toll-like receptor (TLR) pattern recognition receptors (PRRs) to prevent their recognition (or restrict their accessibility), or interfering with signaling downstream of these receptors using toxins and effectors (LaRock and Nizet, 2015). NLR and TLR PRRs detect numerous GAS PAMPs including lipopeptides, lipoteichoic acid, peptidoglycan, CpG-rich DNA, SLO [reviewed in (LaRock and Nizet, 2015)], SIC (Neumann et al., 2021), and the C-type lectin agonist monoglucosyldiacylglycerol (Imai et al., 2018). GAS also encodes additional virulence factors that promote pathogenesis by directly activating immune responses rather than inhibiting them (**Table 1**). The complex and seemingly paradoxical contributions of these proinflammatory virulence factor mechanisms are the focus of this review. Additional pathogens with tropism for the skin or upper respiratory mucosa such as *Staphylococcus aureus* and *Streptococcus pneumoniae* can derive specific benefits from inflammation. We draw parallels with the commonalities in virulence factors and their underlying molecular mechanisms where applicable.

**Table 1:** Predominant GAS virulence factors, their known mechanisms and targets, and their essentiality in models of invasive (intradermal, subcutaneous, or intravenous) or nasopharyngeal (intranasal) infection.

Name	Function	Target(s)	*In vivo* requirement?	
			Invasive	Intranasal
Streptococcal pyrogenic exotoxin (Spe A, C, G, H, I, J, K, L, M, Q, R); SmeZ; SSA	Superantigen (binding activity)	TCR, MHC, potentially co-receptors	N (Walker et al., 2014)	Y (Turner et al., 2012; Kasper et al., 2014)
Streptococcal pyrogenic exotoxin B (SpeB)	Protease	IL-1β, 100+ other host & GAS proteins	N (LaRock et al., 2016)	Y (LaRock et al., 2020)
ScpA	Protease	C5a	N (Walker et al., 2014)	Y (Ji et al., 1997)
Streptokinase (Ska)	Protease	Plasminogen activator	Y (Sun et al., 2004)	?
SpyCEP/ScpC	Protease	IL-8	Y (Zinkernagel et al., 2008)	?
IdeS/Mac	Mimicry, Protease	FcγRIIIB; IgG opsonophagocytis	N (Okumura et al., 2013)	?
Sda1	DNase	NETs	Y (Walker et al., 2007)	Y (Tanaka et al., 2020)
Nga	Glycohydrolase	NAD^+^, unknown other	N (Cole et al., 2010)	?
Streptococcal 5′-nucleotidase A (S5nA)	Hydrolase	Nucleosides (AMP, ADP, ATP)	Y (Soh et al., 2020)	?
Streptolysin O (SLO)	Pore-forming toxin	Epithelial & leukocyte membranes	N (Steer et al., 2009) Y (Timmer et al., 2009)	?
Streptolysin S (SLS)	Pore-forming toxin	Erythrocyte membranes	N (Hall et al., 2004) Y (Betschel et al., 1998)	?
M protein	Adhesion, Sequestration	LL-37, histones, albumin, plasminogen, fibrinogen, CD46, Ig, C3b, factor H, C4b-BP	N (Cole et al., 2010) Y (Ashbaugh et al., 1998)	Y (Anderson et al., 2014)
T antigen	Adherence	Fibronectin, collagen	Y (Chen et al., 2020)	?
Protein S	Sequestration	Erythrocyte fragments	Y (Wierzbicki et al., 2019)	?
SIC	Sequestration	Lysozyme, kininogen, defensins, LL-37, C5b	Y (Frick et al., 2003)	Y (Lukomski et al., 2000)
EndoS	Endo-β-N-acetylglucosaminidase	IgG	N (Sjögren et al., 2011)	?
SpyA	ADP ribosyltransferase	Actin, vimentin, others?	Y (Hoff et al., 2011)	?
SodA	Superoxide dismutase	Reactive oxygen species	Y (Ricci et al., 2002; Janulczyk et al., 2003)	?
HasABC	Hyaluronic acid capsule	Antimicrobials, CD44	N (Henningham et al., 2014)	Y (Cywes et al., 2000)

TCR, T cell receptor; MHC, Major histocompatibility complex; NET; neutrophilic extracellular trap; IL, interleukin; FcγRIIIB, Fcγ receptor type III, also referred to as CD16.

## Roles of Proinflammatory Virulence Factors During Infection and in Disease

Proinflammatory pathways have evolved to initiate and coordinate the immune response against pathogens to protect against infection. Correspondingly, transgenic mice with deficiencies in inflammatory signaling are generally more susceptible to infection, as are humans with immunodeficiencies due to genetic disorders, preexisting conditions, or anti-inflammatory drugs (von Bernuth et al., 2008; Picard et al., 2011; Kidd et al., 2015; LaRock et al., 2016; Rose-John et al., 2017; Wilde et al., 2021). The study of when pathogens instead benefit from inflammation is an emergent field that runs counter to this prevailing paradigm.

During GAS infection of the murine nasopharynx (a model of strep throat), neutrophils, T cells, and the proinflammatory cytokine IL-1β all promote GAS growth (Zeppa et al., 2017; LaRock et al., 2020), despite conventionally having vital antimicrobial roles in immunity and acting to counter GAS invasion at other body sites (LaRock et al., 2016; Pinho-Ribeiro et al., 2018). In antibiotic-treated mice, neutrophils and IL-1β are no longer essential for GAS colonization of the nasopharynx, suggesting this immune axis may be necessary for overcoming interference from antibiotic-sensitive members of the resident microbiota (LaRock et al., 2020). Diverse pathogens including *Salmonella enterica* serovar Typhimurium (Winter et al., 2010; Arpaia et al., 2011), *Helicobacter pylori* (Kusters et al., 2006), *Pseudomonas aeruginosa* (Palomo et al., 2014; Sun et al., 2020), and *Candida albicans* (Jawhara et al., 2008) also subvert inflammation to their advantage, either to disrupt membrane barrier function, promote dissemination, acquire nutrients, or antagonize competing microbes. For the best-characterized example closely related to GAS, *Streptococcus pneumoniae*, inflammation broadly promotes growth and transmission, though T cells, neutrophils, and IL-1β each have specifically antagonistic effects on *S. pneumoniae* growth (Weiser et al., 2018). Thus while several pathogens have a common strategy of subverting inflammation for their benefit, the specific mechanisms and benefits can greatly differ.

The molecular basis underlying the requirement for T cells at the earliest stages of GAS infection is more enigmatic, but later T cells responses are uncoordinated, non-specific, and potentially less effective (Zeppa et al., 2017). Excessive inflammation can promote epitope spreading, whereby increased activation of antigen-presenting cells and T cells leads to broader specificities and increased chance of recognizing self-antigen (Lehmann et al., 1992; Brocke et al., 1993). Recurrent GAS infections drive the generation of autoreactive antibodies that cross-react with heart valve endothelium, lysogangliosides, dopamine receptors, and other human tissues (Cunningham, 2019). These antibodies may give rise to immune sequelae such as acute rheumatic fever (ARF) and rheumatic heart disease (RHD), which account for a majority of annual deaths from GAS (Ralph and Carapetis, 2012); however, the pathogenesis of these conditions remains controversial. A neurological manifestation of these autoreactive antibodies is Sydenham chorea (SC), characterized by an uncontrolled movement of the arms, legs, and facial muscles (Joshi et al., 2015).

The aberrant immune activation GAS has evolved to promote nasopharyngeal infection drives morbidity and mortality when GAS is found in other body sites. Excessive systemic inflammation directly induces death through sepsis, organ failure, disseminated intravascular coagulation, thrombosis, and edema (Cohen et al., 2015). During invasive skin infections like necrotizing fasciitis, inflammation drives localized microvascular thrombosis, tissue hemorrhage, and cell infiltrate, all which further the proinflammatory cycle (Keller et al., 2018). Through these mechanisms, which can limit the perfusion of antibiotics and provide a protective intracellular niche within macrophages, inflammation may also contribute to antibiotic failure (Thulin et al., 2006). In the following sections, we discuss how specific GAS virulence factors that induce inflammation contribute to disease. We focus on those for which inflammation is a specifically beneficial feature for GAS and not only a linked but detrimental consequence ancillary to a secondary function.

## SpeB, a Broad-Spectrum Protease

The Streptococcal pyrogenic exotoxin B (SpeB) is a secreted cysteine protease with broad specificity, high turnover, and which can comprise up to 95% of the total secreted protein that can be detected from GAS (Aziz et al., 2004). In tissue, SpeB cleavage of occludin, E-cadherin, and desmoglein can disrupt tight junctions and may enhance tissue invasion and edema (Sumitomo et al., 2013; Sumitomo et al., 2018). SpeB targets several components of the immune system, including cytokines and chemokines, which may inhibit their signaling (Egesten et al., 2009); and cathelicidin/LL-37, defensins, complement proteins, and immunoglobulins, which may enhance GAS resistance to these immune effector molecules (**Table 1**). Cleavage by SpeB is generally assumed to be exclusively destructive, but SpeB cleavage activates bradykinin (Herwald et al., 1996), the protease-activated receptor PAR-1 (Ender et al., 2013), matrix metalloprotease zymogens (Burns et al., 1996; Tamura et al., 2004), and the proinflammatory cytokine IL-1β (LaRock et al., 2016; LaRock et al., 2020). The pathogenic benefit GAS derives from activating proinflammatory cytokines such as IL-1β requires additional study, but may involve disrupting colonization resistance mediated by the microbiota (LaRock et al., 2020). Conversely, SpeB can cleave and potentially inactivate most GAS virulence factors (Aziz et al., 2004), including those with proinflammatory activities such as SLO, superantigens, and M protein (all discussed in greater detail in following sections). However, this may occur only to a limited degree during infection since both SpeB and the virulence factors it cleaves are essential (Aziz et al., 2004; Cole et al., 2006; Walker et al., 2007; Walker et al., 2014). Nonetheless, SpeB is conserved and promotes GAS survival by intradermal (LaRock et al., 2016), subcutaneous (Ashbaugh et al., 1998; Cole et al., 2006), intraperitoneal (Lukomski et al., 1997; Kuo et al., 1998; Lukomski et al., 1998), and intranasal (Lukomski et al., 1999; Makthal et al., 2018; LaRock et al., 2020) routes in murine experimental infections (**Table 1**).

Some of the underlying cost-benefit of SpeB expression is clear from mutants recovered from natural and experimental infections. These include mutations in the two-component regulatory system CovRS (CsrRS) that lead to constitutive *speB* repression, increased expression of other virulence factors that promote tissue invasion, and shifts in cytokine responses (Li et al., 2014; LaRock et al., 2016; Williams et al., 2021). Development of these mutations is variable between GAS serotypes, and more rarely, other mutations are found to disrupt *speB* expression, such as in the positive regulator *ropB* (Friaes et al., 2015; Feng et al., 2016). CovRS mutants are widely observed in human invasive infection (Chatellier et al., 2000; Kansal et al., 2000; Olsen et al., 2015) and murine models of invasive infection (Engleberg et al., 2001), and less frequently during human pharyngitis (Sumby et al., 2006; Ikebe et al., 2010; Shea et al., 2011) or murine models of pharyngitis (LaRock et al., 2016, Cole et al., 2010; Fiebig et al., 2015; Horstmann et al., 2018; LaRock et al., 2020). CovRS mutants are attenuated in transmission and colonization (Hollands et al., 2010), but can have pathogenic synergy in combination with non-mutant GAS that increases disease severity in murine models of invasive infection (Horstmann et al., 2018).

## Superantigens

**Figure 1:**
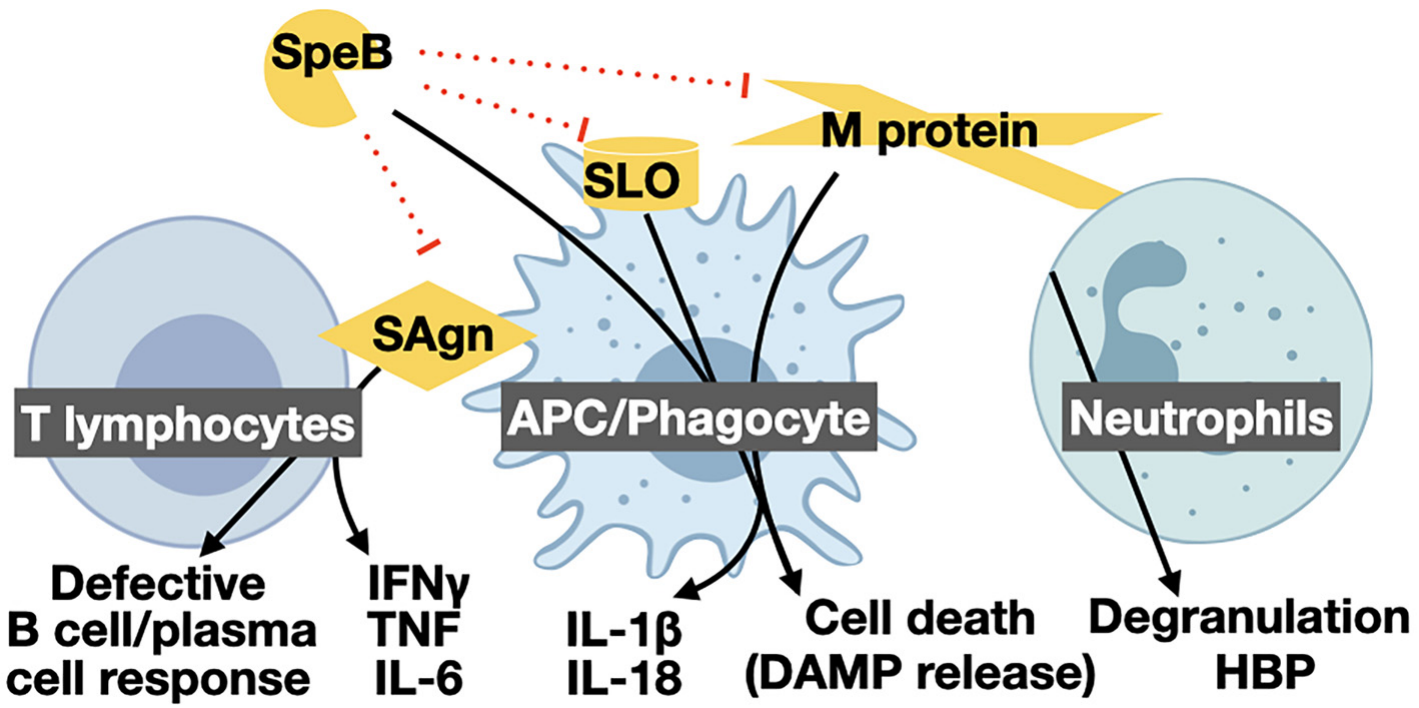
Proinflammatory virulence mechanisms of GAS and their targets. The GAS protease SpeB is directly proinflammatory by activating pro-IL-1β, other host substrates, and inactivating anti-inflammatory GAS effectors. SpeB cleavage of other proinflammatory cytokines, and proinflammatory virulence factors such as superantigens (SAgn), streptolysin O (SLO), and M protein can lead to their inactivation and have anti-inflammatory contributions. Superantigens forcibly bind T lymphocytes and APCs, leading to excessive T cell activation. Activated T cells kill other immune cells and release a “cytokine storm” of IFN-γ, TNF, and IL-6, hallmark of STSS. The pore-forming toxins SLO and streptolysin S (SLS) form large pores in host cells that can lead to the passive release DAMPs, and other cytosolic or organelle-associated proinflammatory compounds, or be detected by the inflammasome to further activate inflammatory cell death by pyroptosis. Proinflammatory effects of SLO can include aiding translocation of the virulence factors Nga. M protein proteolytically released from the GAS surface can similarly form complexes that induce pyroptosis in macrophages or hyper-degranulation by neutrophils. Like other microbes, GAS has numerous TLR agonists that activate proinflammatory regulatory programs [reviewed in (LaRock and Nizet, 2015)]. Created with BioRender.com.

Superantigens are secreted virulence factors that act as T cell mitogens and have strong proinflammatory activity (Proft and Fraser, 2016). Superantigens bypass conventional processing by Antigen Presenting Cells (APCs) and directly bind Major Histocompatibility Complex class II (MHC-II) proteins to the variable β-chain of the T cell Receptor (TCR) (Spaulding et al., 2013). The crosslinking allows for activation of up to 30% of human T cells, compared to the 0.01% typical of an antigen (Lappin and Ferguson, 2009). Mucosal associated invariant T (MAIT) cells are the most responsive to superantigens (Shaler et al., 2017) and major contributors to the “cytokine storm” of pathological and proinflammatory IFN-γ, TNF, IL-6, and IL-1β (**Figure 1**) during STSS (Emgård et al., 2019).

Most GAS encode several superantigens (summarized in **Table 1**), which are typically encoded on mobile genetic elements like lysogenic phage (Ben Zakour et al., 2015). This wide prevalence suggests there is a selective benefit to express superantigens. The other major superantigen-producing pathogen is *Staphylococcus aureus*, which can use superantigens to control bacterial number during nasal colonization of mice (Xu et al., 2015). Superantigens and T cells clearly promote GAS growth in murine nasopharyngeal infections (Kasper et al., 2014; Zeppa et al., 2017). While additional work is required to delineate the pathogenic benefit for GAS, superantigens potentially dampen restrictive innate immune responses by inducing T cell anergy or to increase nutrient availability as a result of inflammation (Sriskandan et al., 2007). Recent research suggests this is true in humans, and shows that superantigens can also direct germinal center follicular helper T cells to kill B cells, and patients with recurrent tonsillitis have smaller tonsil germinal centers and reduced antibody responses (Dan et al., 2019). Thus, superantigens may not only promote infection but also induce host susceptibility to subsequent infection.

## Pore-Forming Toxins

One of the first virulence factors identified in GAS was streptolysin O (SLO), a toxin that forms pores ~30 μm in diameter in the plasma membrane of target cells (Herbert and Todd, 1941). SLO targets immune cells and keratinocytes for translocation of the co-regulated NAD-glycohydrolase toxin Nga (Sumby et al., 2005), which can promote the intracellular survival of GAS (Bastiat-Sempe et al., 2014). Like many other pore-forming toxins, SLO is TLR4 agonist and can induce proinflammatory cytokine expression (Park et al., 2004), and also activates the NLRP3 inflammasome to induce inflammatory cell death by pyroptosis of macrophages (**Figure 1**) and the maturation and release of proinflammatory IL-1β (Timmer et al., 2009). This inflammation may be beneficial for GAS during pharyngitis, where IL-1β promotes GAS growth (LaRock et al., 2020), but may also work to eliminate cells that could restrict GAS growth in other circumstances. During interactions with neutrophils, SLO leads to release of azurocidin that induces edema (Nilsson et al., 2006), while in all cells, the released cytosolic contents are detected by PRRs as damage-associated molecular patterns (DAMPs) that induce further inflammation [Reviewed in (LaRock and Nizet, 2015)]. Other recent work indicates that sub-lethal quantities of SLO and NGA can be anti-inflammatory, leading to degradation of pro-IL-1β and suppress IL-1β activation, suggesting there are more complexities to this immunomodulation yet to discover (Hancz et al., 2017; Hancz et al., 2019; Westerlund et al., 2019).

GAS encodes a second pore-forming toxin, streptolysin S (SLS), which resembles bacteriocins in sequence and function (Nizet et al., 2000). SLS lyses a broad array of cells including erythrocytes, platelets, lymphocytes, and keratinocytes (Ofek et al., 1970), and is responsible for β-hemolysis (**Table 1**). SLS-induced death of keratinocytes can disrupt tissue, promoting lesion formation and dissemination (Sumitomo et al., 2011). SLS also activates the p38 MAPK and NF-kB pathways, broadly inducing of IL-1β, IL-6, and other proinflammatory cytokines (Goldmann et al., 2009) that can be beneficial for GAS at sites where inflammation is beneficial, such as the nasopharynx (LaRock et al., 2020). Lastly, SLS directly activates nociceptor neurons to release the neuropeptide calcitonin gene-related peptide (CGRP), which induces pain and increases necrotizing fasciitis severity (Pinho-Ribeiro et al., 2018).

## M Protein

M protein is the most abundant protein on the GAS surface, forming dimeric coil-coils that extend as hair-like fibrils from the cell wall (Smeesters et al., 2010). There are over 250 allelic variants, each with the variable ability for binding different host factors which include fibrinogen, plasminogen, C4b-binding protein, Protein H, IgA, IgG, LL-37, and the histones contained within antimicrobial neutrophil extracellular traps (**Table 1**). Each of these interactions can promote virulence by both binding host immune effectors and coating the bacterial surface with a barrier of non-immunogenic endogenous proteins. Binding to each of these factors primarily occurs in the variable N-terminal region of M protein; the C-terminus is more conserved and thought to function as a stalk to project these functions distally from the surface (Ghosh, 2018). Few M protein alleles have been comprehensively examined for their host factors they target, but the binding motifs mediating most of these interactions are widespread, with most M alleles predicted to bind several different host factors (Sanderson-Smith et al., 2014).

Surface-anchored M protein is not proinflammatory, however, some alleles of M protein can also be released and gain drastically altered activities (Elliott, 1945; Berge and Bjorck, 1995; Herwald et al., 2004; LaRock et al., 2015; Dohrmann et al., 2017; Valderrama et al., 2017). Both neutrophil proteases (Herwald et al., 2004) and SpeB (Berge and Bjorck, 1995) may be involved in M protein release. Soluble M protein has greater availability as an antigen, a PRR agonist, and can form toxic aggregates with fibrinogen to induce cell death, neutrophil degranulation, and vasculature leakage responses (Herwald et al., 2004; Soehnlein et al., 2008; Macheboeuf et al., 2011; Hertzén et al., 2012; Walker et al., 2014). Some M protein alleles may also have weak T cell mitogen activity (Påhlman et al., 2007). Binding of M protein to platelets leads to their activation and thrombosis (Horváth et al., 2004; Shannon et al., 2007; Palm et al., 2019). Lastly, M protein is a major agonist of TLR2, inducing expression of the numerous proinflammatory molecules regulated by NF-kB (Pahlman et al., 2006), and activates the NLRP3 inflammasome, resulting in proinflammatory cell death by pyroptosis (Valderrama et al., 2017). As with pore-forming toxins, these proinflammatory activities contribute to the pathology and complications of infection, but it is not clear whether they are required for virulence.

## Potential for Therapeutics to Manage Infection

There is no GAS vaccine after 100 years of research. Ongoing preclinical and clinical work focus on the immunodominant, but variable, M protein, the conserved group A carbohydrate, and multi-valent vaccines against major GAS virulence factors [reviewed in (Dale et al., 2016)]. Invasive infections have a mortality rate upwards of 20% within seven days of the onset, and treatments include antibiotic therapy and surgical debridement of infected necrotic tissue (Wilkins et al., 2017). GAS remains penicillin sensitive (Vannice et al., 2020), and clindamycin is recommended for severe infections and patients with penicillin allergies (Sriskandan et al., 1997; Mascini et al., 2001; Steer et al., 2012). Macrolide resistance is increasingly prevalent (Silva-Costa et al., 2015; Fyfe et al., 2016; Michos et al., 2016), but tedizolid and linezolid may be used instead (Bryant et al., 2020). Despite the availability of antibiotics to treat infection, antibiotic monotherapy can fail to eradicate GAS during pharyngitis or invasive disease (Kim and Kaplan, 1985; Steer et al., 2012). Antibiotic inefficacy is multifactorial and can be due to bacterial tolerance, reservoirs of protected intracellular bacteria, failure of the drug to reach the infection site due to tissue necrosis and thrombosis, and the rapid progression of disease (Spinaci et al., 2006; Hertzén et al., 2010; Fischetti and Dale, 2016). Below, we will discuss recent developments in therapeutics based on targeting inflammation and proinflammatory virulence factors.

Intravenous immunoglobulin (IVIG) are non-specific antibodies pooled from human donors. As an adjunctive therapy, IVIG may decrease morbidity by not only opsonizing the bacteria, but neutralizing GAS exotoxins (Norrby-Teglund et al., 2005; Low, 2013). In murine models, IVIG treatment in conjunction with penicillin and clindamycin was successful at modulating the systemic inflammatory response as well as increasing bacterial killing (Sriskandan et al., 2006). In human trials, IVIG treatment is associated with a 20-30% increase in survival during STSS (Linnér et al., 2014), but this may have lessor benefit in children (Shah et al., 2009). The use of IVIG for the treatment of GAS disease is an area of active research, but shows promise in safety and efficacy, though may be cost-prohibitive in many regions where the health burden is highest (Williams et al., 2016).

Several FDA-approved drugs have potential for off-label use during GAS disease. The HIV protease inhibitor nelfinavir inhibits streptolysin S, limiting its pro-virulence and proinflammatory activities (Maxson et al., 2015). Excessive inflammation during STSS and other conditions is harmful, and therapeutics targeting inflammation may also have therapeutic benefit. During invasive infections, the opioid-derivative cough suppressant dextromethorphan may prolong survival through its anti-inflammatory activities (Li et al., 2011; Chen et al., 2018; Zhou et al., 2019). Drugs that block nociceptor signaling also promote beneficial immune responses (Pinho-Ribeiro et al., 2018). Lastly, inhibiting protein synthesis can block production of SpeA, the nuclease Sda1, SLO, and other toxins (Herbert et al., 2001; Mascini et al., 2001; Goscinski et al., 2006; Andreoni et al., 2017). Thus, antibiotics like clindamycin may have therapeutic benefits in addition to direct killing of GAS.

## Concluding Remarks

As an obligate human pathogen GAS is highly adapt at manipulating human innate and adaptive immune responses. This often serves to resist immune effectors, mask the pathogen, and subvert immune signaling (**Table 1**), but GAS also induces and thrives off the activation of other immune processes (**Figure 1**). New insights into the molecular mechanisms involved may come from forward genetic screens based on high-throughput sequencing of transposon mutant libraries, which have already provided new insights into regulatory, metabolic, and antimicrobial resistance mechanisms that contribute to fitness during infection (Le Breton et al., 2015; Zhu et al., 2019). The complicated relationship of GAS with inflammation can underlie infectious risk with some anti-inflammatory therapeutics, like inhibitors of IL-1β, IL-6, or TNF, but may provide opportunities for treatments with other immune-targeted drugs.

Host-directed molecules based on a better understanding of the role of inflammation in GAS pathogenesis hold hope for future therapeutics. Debilitating and difficult-to-treat disease manifestations following GAS infection like rheumatic heart disease, scarlet fever, toxic shock syndrome, and post-streptococcal glomerulonephritis result from aberrant and excessive immune responses during and after infection and can potentially be treated through immunomodulation. Since many GAS deaths are from immune-mediated complications, knowledge of which GAS factors induce inflammation, and which inflammatory pathways drive pathology in immune disease, will be essential for these strategies. Careful dissection of these processes will be essential to develop adjunctive therapies to reduce the significant morbidity and mortality caused by GAS.

## Author Contributions 

All authors listed have made a substantial, direct, and intellectual contribution to the work, and approved it for publication.

## Funding

Work in the LaRock lab is supported by NIH/NIAID AI153071. The funders had no role in study design, data collection and analysis, decision to publish, or preparation of the manuscript.

## Conflict of Interest

The authors declare that the research was conducted in the absence of any commercial or financial relationships that could be construed as a potential conflict of interest.
